# Associations between cochlear electrophysiology, emotional health, and sleep quality in adults with tinnitus: a comprehensive analysis

**DOI:** 10.3389/fpsyt.2025.1721036

**Published:** 2026-01-30

**Authors:** Ye Liu, Lihao Bao, Xiaojiong Mao, Kaixing Shao, Guihua Xia, Shaosheng Liu, Ke Ji

**Affiliations:** 1 Beilun Branch, First Affiliated Hospital of Zhejiang University School of Medicine, Ningbo, Zhejiang, China; 2 Department of Otolaryngology, Ningbo Beilun People’s Hospital, Ningbo, Zhejiang, China

**Keywords:** auditory brainstem response, depression, electrocochleography, emotion regulation, sleepdisorders, tinnitus

## Abstract

**Introduction:**

Tinnitus is commonly accompanied by emotional distress and sleep disturbances, yet the extent to which these characteristics relate to cochlear electrophysiologic findings remains unclear. This study examined associations between electrophysiologic measures and emotional and sleep parameters in adults with subjective tinnitus.

**Methods:**

This retrospective study included 120 adults with tinnitus. Data collected included demographics, cochlear electrophysiologic measures (electrocochleography and auditory brainstem response), emotional characteristics (perceived stress, depressive symptoms, anxiety, emotion regulation), and sleep parameters (sleep quality, insomnia severity, daytime sleepiness). Correlation analyses and multivariate regression models were applied.

**Results:**

The mean age of the cohort was 62.35 years (SD 9.45), and 64.17% were male. A very weak negative correlation was observed between depressive symptoms and Wave III latency (r = –0.196, P = 0.03), but the small magnitude suggests minimal explanatory value. The summating potential/action potential ratio was not significantly correlated with sleep quality (r = –0.181, P = 0.05). In multivariate models, anxiolytic use was associated with a lower risk of poor sleep (adjusted odds ratio [aOR] = 0.262; 95% confidence interval [CI]: 0.078–0.881; P = 0.030), whereas antidepressant use was associated with a higher risk (aOR = 2.628; 95% CI: 1.027–6.724; P = 0.044). For insomnia, higher pure-tone average thresholds (aOR = 0.948 per dB; 95% CI: 0.906–0.992; P = 0.007) and hearing aid use (aOR = 3.396; 95% CI: 1.085–10.623; P = 0.036) were significant determinants. The only significant factor associated with quality of life was tinnitus duration, with longer duration associated with lower WHOQOL-BREF scores (β = –2.74; P = 0.020). No electrophysiologic parameter demonstrated significant associations in the multivariate models.

**Conclusion:**

Within the constraints of this study, cochlear electrophysiologic measures showed limited observable associations with emotional or sleep characteristics. Factors related to hearing status and medication use demonstrated stronger statistical associations with sleep outcomes, while tinnitus duration was linked to quality-of-life scores. These findings contribute to the growing body of descriptive evidence on tinnitus-related characteristics, although further research with larger cohorts and longitudinal designs is needed to clarify these relationships.

## 1 Introduction

Tinnitus—defined as the perception of sound in the absence of an external auditory stimulus—affects approximately 10–15% of adults and is frequently associated with reduced quality of life (1). Emotional distress is highly prevalent among individuals with tinnitus, with prior studies documenting elevated levels of stress (2), depression (3), and anxiety (3), all of which can exacerbate patients’ subjective symptom burden (4). Sleep disturbances, reported in up to 70% of tinnitus patients (5), further contribute to distress and functional impairment. Although these domains—emotional health, sleep quality, and tinnitus perception—are frequently interrelated in clinical practice, the neurobiological pathways that link them remain incompletely understood.

Objective auditory measures such as electrocochleography (ECochG) and auditory brainstem response (ABR) are commonly used to assess peripheral and brainstem auditory pathway function (6–9). These measures are grounded in established auditory physiology: the SP/AP ratio reflects cochlear hair cell and endolymphatic function (10), whereas ABR Wave I and Wave III latencies index auditory nerve and pontine transmission timing (11). Although aberrant auditory processing has been reported in subsets of tinnitus patients (7–9), prior work has not demonstrated consistent associations between ECochG or ABR parameters and subjective symptoms such as emotional distress or sleep impairment. For example, Kehrle et al. (12) reported ABR alterations in tinnitus but found no relationship with depression or anxiety scores, illustrating that electrophysiologic abnormalities may occur independently of psychological symptom severity.

Research on tinnitus-related emotional distress (13), sleep disturbances (14, 15), and auditory electrophysiology (16) has largely progressed in parallel rather than in an integrated manner. Most electrophysiology studies have focused on auditory pathway structure and timing without incorporating psychological or sleep measures, whereas studies examining emotional or sleep outcomes seldom include ECochG or ABR testing (17).

Tinnitus-related burden spans multiple functional levels: cochlear and brainstem activity measured through electrophysiologic testing (18), perceptual–emotional experiences such as stress, anxiety, and depression (13), behavioral manifestations including sleep disruption (19), and higher-order regulatory processes such as cognitive reappraisal and expressive suppression (20). These levels are clinically interconnected, as emotional distress can heighten the salience of tinnitus and influence sleep quality (19), while coping and emotion regulation styles may shape how strongly the percept intrudes on daily functioning (20). Considering these domains together therefore provides a coherent view of how distinct aspects of tinnitus burden may converge or diverge within the same individual (21). This conceptual structure motivates the present study’s descriptive aim: to determine whether these domains display measurable correspondence or remain largely independent, thereby clarifying which assessments offer complementary clinical information (13, 18).

Given this fragmentation in the literature, a unified examination of auditory physiology, emotional characteristics, sleep quality, and emotion regulation may help clarify which symptom domains tend to cluster together and which remain distinct. Such an approach does not aim to test mechanistic pathways or causal models but rather to provide a descriptive assessment of whether these commonly evaluated domains show measurable correspondence within the same patient cohort. Understanding these relationships may assist clinicians in determining which assessments offer complementary information and which domains may not meaningfully align with electrophysiologic findings.

This study therefore seeks to characterize the degree of correspondence among cochlear electrophysiologic parameters (ECochG and ABR), emotional and psychological features (stress, depression, anxiety), sleep-related measures, and emotion regulation strategies in patients with tinnitus. By evaluating these domains concurrently, we aim to clarify whether objective auditory measures relate to psychological or sleep outcomes and to identify potential areas where routine screening may—or may not—provide additional clinical value.

## 2 Methods

### 2.1 Study design and setting

This retrospective observational study analyzed prospectively collected data from adult patients with subjective tinnitus who attended the Department of Otolaryngology at Ningbo Beilun People’s Hospital between January 2024 and January 2025. The study was conducted to examine whether psychological disturbances—such as stress, depression, anxiety, emotion regulation patterns, and sleep impairment—show measurable associations with cochlear electrophysiologic parameters. All procedures complied with the Declaration of Helsinki. Ethical approval was granted by the Institutional Ethical Review Board of Ningbo Beilun People’s Hospital (Approval number: 2024LP035). Written informed consent was obtained from all participants.

### 2.2 Study population

A total of 120 consecutive adults diagnosed with subjective tinnitus were recruited from the outpatient otology clinic.

Inclusion criteria:

- Age ≥ 18 years.
- Diagnosis of subjective tinnitus confirmed through clinical otologic evaluation, audiometry, and exclusion of alternative auditory symptoms (e.g., pulsatile vascular tinnitus, objective middle ear sources).
- Ability to provide informed consent and complete study questionnaires.

Exclusion criteria:

- History of Meniere’s disease, vestibular schwannoma, or otosclerosis.
- Major psychiatric illness documented in the electronic medical record, including schizophrenia, bipolar disorder, psychotic disorders, or severe major depressive disorder requiring psychiatric care.
- Use of ototoxic medications within the past 6 months.
- Cognitive impairment interfering with questionnaire completion.

Identification of psychiatric illness:

Diagnoses were extracted from the hospital’s electronic medical record, which uses ICD-10–based coding. All psychiatric diagnoses were made by board-certified psychiatrists or neurologists as part of routine clinical care. These diagnoses—not questionnaire scores—served as the basis for exclusion. Screening instruments (BDI-II, GAD-7, PSS) were used only to assess symptom severity, not to establish or exclude psychiatric diagnoses.

### 2.3 Data collection

All participants underwent comprehensive audiological, electrophysiological, and psychological assessments. Data were collected using standardized questionnaires and objective tests. A single researcher administered all tests to minimize variability in data collection. The data collection process followed a consistent sequence: each patient first completed baseline demographic and tinnitus-related questionnaires, followed by audiometric and electrophysiologic testing (ECochG, ABR, and OAE). Subsequently, standardized self-report questionnaires assessing stress, depression, anxiety, emotion regulation, and sleep quality were administered in a dedicated counseling session on the same day.

#### 2.3.1 Baseline clinicodemographic data

Collected variables included age, sex, tinnitus duration, tinnitus laterality, hearing thresholds (dB), pure-tone audiometry, and tinnitus severity. Tinnitus severity was evaluated using the Tinnitus Handicap Inventory (THI), scored from 0 to 100 and interpreted as: 0–16 slight/no handicap, 18–36 mild, 38–56 moderate, 58–76 severe, and 78–100 catastrophic.

#### 2.3.2 Cochlear electrophysiologic measurements

Electrophysiologic measurements were obtained using Electrocochleography (ECochG) and Auditory Brainstem Response (ABR) testing.

- ECochG readings were classified into two categories: normal and abnormal.
- ABR testing was performed using a Neuro-Audio ABR System at an intensity of 80 decibels in normal Hearing Level (dBnHL). Latencies for ABR Wave I, III, and V were recorded. The SP/AP ratio was calculated from the summating potential to action potential measurements.
- Otoacoustic Emission (OAE) testing was conducted at 500 Hz to assess outer hair cell function, and results were recorded as either present or absent.

#### 2.3.3 Emotional and psychological assessments

Psychological and emotional distress were assessed using validated Chinese-language versions of the following scales:

- Perceived Stress Scale (PSS): A 10-item scale used to measure the perception of stress. Total scores range from 0 to 40, with higher scores indicating greater perceived stress. Patients with scores ≥ 27 can be categorized as experiencing high stress (22).
- Beck Depression Inventory-II (BDI-II): A 21-item self-report inventory measuring depressive symptoms. Scores range from 0 to 63, with higher scores reflecting more severe depression. Participants with scores ≥ 20 can be categorized as having clinically significant depression (23).
- Generalized Anxiety Disorder-7 (GAD-7): A 7-item scale used to screen for anxiety disorders. Scores range from 0 to 21, with scores ≥ 10 indicative of moderate to severe (clinically significant) anxiety (24).
- Emotion Regulation Questionnaire (ERQ): A 10-item scale measuring two emotion regulation strategies: cognitive reappraisal and expressive suppression. The cognitive reappraisal domain is scored between 6 and 42, with scores above 26 being indicative of high cognitive reappraisal. The expressive suppression domain was scored from 4 to 28, with scores of 16 or above indicating high expressive suppression (25).

Each scale measures a distinct psychological construct; they were selected due to their established psychometric validity and widespread use in auditory and behavioral research.

#### 2.3.4 Sleep characteristics and quality of life

Sleep disturbances were evaluated using the following standardized instruments:

- Pittsburgh Sleep Quality Index (PSQI): A self-rated questionnaire assessing sleep quality over the past month. Scores > 5 indicate poor sleep quality (26).
- Insomnia Severity Index (ISI): A 7-item scale evaluating the severity of insomnia. Scores ≥ 15 suggest clinically significant insomnia (27).
- Epworth Sleepiness Scale (ESS): An 8-item scale measuring daytime sleepiness. Patients with scores ≥ 11 can be considered to have excessive daytime sleepiness (28).
- World Health Organization Quality of Life (WHOQoL-BREF): A 26-item scale assessing overall quality of life across four domains: physical health, psychological health, social relationships, and environment. We analyzed the overall score, with higher scores indicating better quality (29).

### 2.4 Statistical analysis

All analyses were performed using Stata version 18 (StataCorp, College Station, TX). A two-sided p-value < 0.05 was considered statistically significant. Descriptive statistics were used to summarize demographic, clinical, electrophysiologic, psychological, and sleep-related variables. Continuous variables were reported as means and standard deviations or medians with interquartile ranges where appropriate, and categorical variables were expressed as frequencies and percentages. Pearson correlation coefficients were used to examine the association between continuous electrophysiologic parameters and psychological or sleep measures.

Regression analyses proceeded in two stages. First, univariate regression models were fitted for each outcome of interest, including high stress, clinically significant depression, moderate-to-severe anxiety, poor sleep quality, clinically significant insomnia, excessive daytime sleepiness, and overall quality of life. Univariate logistic regression was applied to binary outcomes, whereas linear regression was used for the WHOQOL-BREF score. Variables demonstrating an association with the outcome at a significance threshold of p < 0.10 in the univariate models were subsequently entered into multivariate models.

Multivariate logistic and linear regression models were then constructed using the selected variables. Prior to model fitting, multicollinearity was assessed using the Variance Inflation Factor (VIF), and predictors with VIF values greater than 10 were excluded to avoid collinearity effects. Model diagnostics included evaluation of Akaike Information Criterion (AIC) and Bayesian Information Criterion (BIC) values, while goodness of fit for logistic regression models was assessed using the Hosmer–Lemeshow test. All final models report odds ratios for logistic regressions and beta coefficients for linear regressions, each accompanied by 95% confidence intervals.

## 3 Results

### 3.1 Baseline data

This study enrolled 120 adult patients diagnosed with tinnitus, with a mean age of 62.35 ± 9.45 years, predominantly male (64.17%). The average hearing threshold was 67.49 ± 11.50 dB, and the mean tinnitus duration was 2.39 ± 0.94 years. Tinnitus was more commonly unilateral (75%), with 79.17% of patients not reporting hyperacusis. Tinnitus severity was categorized as moderate in 39.17% and severe in 20.83%, with catastrophic cases being rare (0.83%). The majority of patients used hearing aids (80.83%), while tinnitus masking devices were utilized by only 10% (**Table 1**)​.

**Table 1:** Baseline clinicodemographic and tinnitus-related data.

Variable	Measure	Min	Max
Age			
Mean (SD)	62.35 (9.45)	27.9	85.4
Gender - no. (%)			
Female	43 (35.83%)	–	–
Male	77 (64.17%)	–	–
Hearing threshold (dB)			
Mean (SD)	67.49 (11.50)	36	93
Pure-Tone Audiometry (dB)			
Mean (SD)	58.51 (9.09)	31	83
Tinnitus Data			
Disease duration - yr.			
Mean (SD)	2.39 (0.94)	0.1	4.9
Frequency (Hz)			
Mean (SD)	2900.83 (502.18)	1800	4000
Loudness (dB)			
Mean (SD)	13.33 (2.07)	7	19
Severity - no. (%)			
Slight/no handicap	16 (13.33%)	–	–
Mild	27 (22.50%)	–	–
Moderate	47 (39.17%)	–	–
Severe	25 (20.83%)	–	–
Catastrophic	1 (0.83%)	–	–
Missing value	4 (3.33%)		
Laterality - no. (%)		–	–
Unilateral	90 (75%)	–	–
Bilateral	30 (25%)	–	–
Hyperacusis - no. (%)			
No	95 (79.17%)	–	–
Yes	25 (20.83%)	–	–
Use of Hearing Aids - no. (%)			
No	23 (19.17%)	–	–
Yes	97 (80.83%)	–	–
Tinnitus Masking Device - no. (%)			
No	108 (90%)	–	–
Yes	12 (10%)	–	–
Medications used - no. (%)			
Sleep medications	93 (77.50%)	–	–
Anti-depressants	68 (56.67%)	–	–
Anxiolytics	88 (73.33%)	–	–

no.: frequency; SD: standard deviation.

### 3.2 Cochlear electrophysiologic findings

Electrocochleography (ECochG) revealed abnormal readings in 43.33% of patients, while otoacoustic emissions at 500 Hz were absent in a similar proportion (43.33%). Auditory brainstem response (ABR) testing demonstrated mean latencies of 2.08 ± 0.28 ms, 4.34 ± 0.37 ms, and 6.00 ± 0.53 ms for Waves I, III, and V, respectively, with an average SP/AP ratio of 0.6 ± 0.2 (**Supplementary Table S1**)​.

### 3.3 Emotional and psychological profiles

patients exhibited significant emotional distress, with 40.83% classified as stressed (PSS ≥ 27) and 41.67% demonstrating clinically significant depressive symptoms (BDI-II ≥ 20). Moderate to severe anxiety was observed in 57.50% of participants (GAD-7 ≥ 10). Regarding emotion regulation strategies, high cognitive reappraisal and expressive suppression scores were noted in 73.33% and 68.33% of patients, respectively (**Supplementary Table S2**)​.

### 3.4 Sleep disturbances and quality of life

Sleep quality was notably impaired, with 70% of participants reporting poor sleep quality (PSQI > 5), while 40.83% met criteria for clinically significant insomnia (ISI ≥ 15). Excessive daytime sleepiness (ESS ≥ 11) was less prevalent, affecting 17.5%. The mean sleep duration was 5.03 ± 1.77 hours, with a mean sleep latency of 1.45 ± 0.85 hours. Frequent awakenings (mean = 1.52 ± 0.97 per night) further disrupted sleep. Quality of life assessments indicated a mean WHOQoL-BREF score of 67.49 ± 12.13, reflecting moderate impairment (**Supplementary Table S3**)​.

### 3.5 Correlation and regression analyses

No substantial correlations were identified between ABR or SP/AP ratios and emotional or psychological variables. A very weak negative correlation was observed between Wave III latency and depressive symptoms (r = –0.1958, P = 0.03), and the SP/AP ratio showed only a minimal, borderline correlation with sleep quality (r = –0.1808, P = 0.05). These small effect sizes indicate limited evidence of any meaningful association (**Table 2**)​.

**Table 2:** Correlation analysis between cochlear electrophysiologic parameters and emotional/psychological outcomes.

Variables	ABR	SP/AP	Wave I latency	Wave III latency	Wave V latency	PSS	BDI-II	GAD-7	ERQ	PSQI	ISI	ESS	WHOQoL
ABR	1												
*P-value*	–												
SP/AP ratio	*r* = 0.0765	1											
*P-value*	0.41	–											
Wave I Latency	r=0.0048	r=0.0667	1										
*P-value*	0.96	0.47	–										
Wave III Latency	r=-0.0533	r=-0.1324	r=-0.0551	1									
*P-value*	0.56	0.15	0.55	–									
Wave V Latency	r=0.0019	r=0.007	r=0.0456	r=0.0697	1								
*P-value*	0.98	0.94	0.62	0.45	–								
PSS score	r=-0.013	r=0.0219	r=0.0449	r=0.0981	r=0.0019	1							
*P-value*	0.89	0.81	0.63	0.29	0.98	–							
BDI-II	r=0.1602	r=0.0892	r=0.0237	r=-0.1958	r=0.0558	r=-0.0912	1						
*P-value*	0.08	0.33	0.80	0.03	0.55	0.32	–						
GAD-7	r=-0.006	r=-0.0725	r=-0.1477	r=0.1131	r=0.018	r=-0.1465	r=-0.0013	1					
*P-value*	0.95	0.43	0.11	0.22	0.85	0.11	0.99	–					
ERQ	r=0.0479	r=-0.0028	r=0.0001	r=-0.0091	r=-0.0852	r=0.0046	r=0.1117	r=0.1274	1				
*P-value*	0.60	0.98	1.00	0.92	0.35	0.96	0.22	0.17	–				
PSQI	r=0.0757	r=-0.1808	r=0.0751	r=-0.0447	r=0.0502	r=-0.1125	r=0.1225	r=0.0416	r=-0.0426	1			
*P-value*	0.41	0.05	0.42	0.63	0.59	0.22	0.18	0.65	0.64	–			
ISI	r=0.068	r=-0.0851	r=-0.0539	r=-0.0637	r=-0.0126	r=-0.1233	r=0.1441	r=0.0419	r=-0.0037	r=0.122	1		
*P-value*	0.46	0.36	0.56	0.49	0.89	0.18	0.12	0.65	0.97	0.18	–		
ESS	r=0.0448	r=0.0242	r=-0.1076	r=0.088	r=-0.0421	r=-0.1395	r=0.05	r=-0.0197	r=-0.1077	r=0.0603	r=0.0335	1	
*P-value*	0.63	0.79	0.24	0.34	0.65	0.13	0.59	0.83	0.24	0.51	0.72	–	
WHOQoL	r=0.0001	r=0.0334	r=0.0011	r=0.0678	r=0.0691	r=0.0048	r=0.0784	r=-0.0167	r=0.025	r=0.0995	r=0.0474	r=0.1539	1
*P-value*	1.00	0.72	0.99	0.46	0.45	0.96	0.39	0.86	0.79	0.28	0.61	0.09	–

ABR, Auditory Brainstem Response; SP/AP ratio, Summating potential to action potential ratio; PSS, Perceived Stress Scale; BDI-II, Beck Depression Index II; GAD-7, General Anxiety Disorder-7; ERQ, Emotion Regulation Questionnaire.; PSQI, Pittsburgh Sleep Quality Index; ISI, Insomnia Severity Scale; ESS, Epworth Sleepiness Sale; WHOQOL-BREF, World Health Organization Quality of Life – BREF; SDSS, Social Functioning Deficit Screening Scale

**Figure 1:**
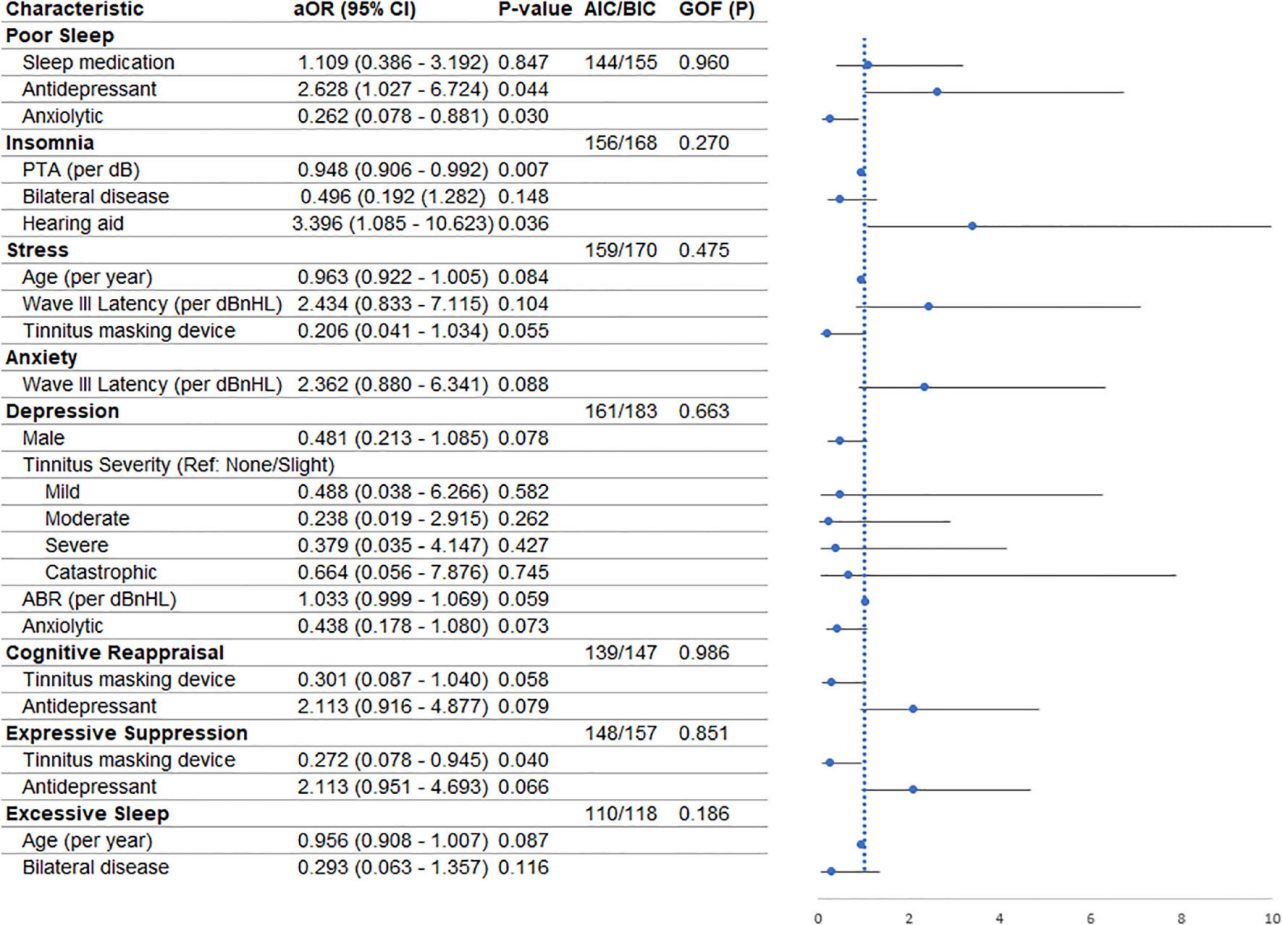
Forest plot showing adjusted odds ratios (aORs) with 95% confidence intervals (CIs) for factors associated with sleep-related outcomes, psychological symptoms, and coping strategies in multivariable logistic regression models. Model fit indices, including Akaike Information Criterion (AIC) and Bayesian Information Criterion (BIC), and goodness-of-fit (GOF) p-values are reported for each model. The vertical dashed line indicates the null value (aOR = 1).

Univariate logistic regression results are presented in **Supplementary Tables S4–S12**, and variables with P < 0.10 were included in the multivariate models (**Figure 1**).

#### 3.5.1 Poor sleep

After controlling for confounders, anxiolytics were associated with a lower risk of poor sleep (aOR = 0.262; 95% CI: 0.078–0.881; P = 0.030), while antidepressants were associated with a higher risk (aOR = 2.628; 95% CI: 1.027–6.724; P = 0.044). Sleep medication use was not significantly associated (P = 0.807).

#### 3.5.2 Insomnia

After adjustment, higher pure-tone average (PTA) thresholds were associated with a lower risk of insomnia (aOR = 0.948 per dB; 95% CI: 0.906–0.992; P = 0.007). Hearing aid use was associated with a higher risk of insomnia (aOR = 3.396; 95% CI: 1.085–10.623; P = 0.036). Bilateral tinnitus was not significantly associated (P = 0.148).

#### 3.5.3 Stress

None of the included factors—age, Wave III latency, or use of tinnitus masking devices—were significantly associated with stress (all P ≥ 0.05).

#### 3.5.4 Anxiety

Wave III latency was the sole variable meeting the inclusion cutoff (p < 0.10) in the univariate mode. However, it was not significantly associated with anxiety (P = 0.088).

#### 3.5.5 Depression

None of the variables retained significance in the adjusted model, including sex, tinnitus severity categories, ABR intensity (P = 0.059), or anxiolytic use (P = 0.073).

#### 3.5.6 Cognitive reappraisal

Neither tinnitus masking devices (P = 0.058) nor antidepressants (P = 0.079) were significantly associated with high cognitive reappraisal.

#### 3.5.7 Expressive suppression

After adjustment, use of tinnitus masking devices was associated with a lower risk of high expressive suppression (aOR = 0.272; 95% CI: 0.078–0.945; P = 0.040). Antidepressant use was not significantly associated (P = 0.066).

#### 3.5.8 Excessive daytime sleepiness

Neither age nor bilateral disease was significantly associated with excessive daytime sleepiness (both P ≥ 0.05).

#### 3.5.9 Quality of life

After controlling for age and other included variables, tinnitus duration was the only significant determinant of WHOQOL-BREF total score. Longer tinnitus duration was associated with lower quality-of-life scores (β = –2.74; P = 0.020; 95% CI: –5.04 to –0.45). No audiologic, electrophysiologic, or psychological measures demonstrated significant associations with quality of life.

Goodness-of-fit (GOF) values indicated acceptable model fit for all multivariate analyses.

## 4 Discussion

Tinnitus is widely recognized as a multidimensional condition with substantial effects on emotional well-being (13), sleep quality (30), and overall quality of life (31). In this cohort of 120 adults with subjective tinnitus, emotional distress and sleep impairment were common, whereas only limited and weak associations were observed between cochlear electrophysiologic measures and psychological or sleep-related parameters. By integrating audiologic, electrophysiologic, psychological, and sleep assessments, this study provides a comprehensive profile of symptom burden, although the strength and pattern of associations highlight the complexity and heterogeneity of tinnitus rather than a unified mechanistic pathway.

### 4.1 Principal findings

Three main observations emerged. First, emotional and sleep disturbances were highly prevalent, with more than half of participants reporting clinically relevant anxiety, depressive symptoms, or poor sleep, consistent with prior research (32). Second, electrophysiologic parameters (ECochG SP/AP ratio, ABR latencies) showed only weak correlations with psychological or sleep variables, and none retained significance after adjustment. The only correlation meeting statistical significance—between ABR Wave III latency and depressive symptoms—had a very small effect size (r≈–0.20), underscoring the minimal explanatory contribution of early auditory pathway measures. Third, multivariate analyses identified non-electrophysiologic variables associated with sleep or quality-of-life outcomes, including PTA thresholds, hearing-aid use, anxiolytic and antidepressant exposure, and tinnitus duration. None of the emotional distress measures nor electrophysiologic parameters were independently associated with stress, anxiety, depression, excessive daytime sleepiness, or emotion-regulation outcomes.

### 4.2 Comparison with existing literature

Previous studies have reported electrophysiologic abnormalities in subsets of tinnitus patients (33, 34), but the magnitude and clinical relevance of these findings have varied. The weak associations observed in this study align with reports showing that electrophysiologic parameters account for minimal variance in psychological outcomes.

Several mechanistic frameworks help contextualize these null results. Contemporary tinnitus models—including central gain enhancement, limbic–auditory coupling, and attentional salience mechanisms—emphasize alterations in central neural processing, rather than abnormalities in peripheral or early brainstem responses detectable through ECochG or ABR (35, 36). These models propose that emotional and cognitive influences on tinnitus distress arise from dysregulated corticolimbic networks (e.g., amygdala–hippocampal–prefrontal interactions) (37, 38) and heightened sensory precision or salience attribution (39), rather than from changes in cochlear or lower brainstem physiology (40). The absence of significant associations in this cohort is therefore consistent with the possibility that the psychological and sleep-related burden of tinnitus reflects central processing mechanisms not captured by peripheral or subcortical electrophysiologic testing.

Sleep disturbances were also common, consistent with previous reports (3, 30, 41, 42). The lack of association between sleep outcomes and electrophysiology supports prior work showing that sleep impairment in tinnitus is more closely related to emotional distress, hyperarousal, and disease severity (43, 44) than to cochlear or brainstem dysfunction. This aligns with the broader understanding that tinnitus-related insomnia reflects heightened arousal and limbic activation rather than abnormalities in sensory transmission (45, 46). Associations between insomnia risk and PTA thresholds or hearing-aid use may reflect behavioral or perceptual factors—such as amplification-related awareness, sleep-environment variables, or chronic disease burden—rather than electrophysiologic abnormalities.

Emotion-regulation strategies (cognitive reappraisal, expressive suppression) showed no significant associations with tinnitus severity, electrophysiology, or emotional distress. Although emotion regulation has been proposed as a contributor to tinnitus-related distress (47), null findings in this cohort are consistent with literature demonstrating substantial heterogeneity in psychological adaptation among tinnitus patients (48). Additionally, ERQ scores reflect higher-order cognitive styles rather than perceptual or sensory-modulatory processes (49, 50), which may explain their independence from electrophysiologic measures. Reduced variability in ERQ scores in an older sample, possible cultural influences on self-reported regulatory style (51, 52), and potential ceiling/floor effects may also contribute. Importantly, ERQ domains assess broad regulatory tendencies, not tinnitus-specific coping strategies, which may make them less sensitive to tinnitus severity or auditory physiology.

### 4.3 Clinical implications

Given the cross-sectional design and the weak associations observed, the findings do not support the use of ECochG or ABR as indicators of emotional distress or sleep impairment in tinnitus patients. While electrophysiologic testing remains valuable for auditory assessment, its relevance for psychological or sleep-related evaluation appears limited in this population. Sleep-related associations with PTA or hearing-aid use should be interpreted cautiously, as they likely reflect behavioral, perceptual, or disease-related factors rather than electrophysiologic abnormalities. Similarly, associations with anxiolytic or antidepressant use may reflect underlying psychiatric conditions rather than medication effects.

Tinnitus duration was the only determinant of quality-of-life scores, consistent with prior studies reporting cumulative burden with chronicity (53). Clinical assessment should therefore prioritize psychological evaluation, sleep assessment, and audiologic review rather than relying on electrophysiologic measures to infer emotional or sleep status.

### 4.4 Limitations and future directions

This study has several limitations. Its cross-sectional design precludes causal inference. Reliance on clinical documentation for psychiatric exclusions introduces a risk of misclassification, as some participants may have had undiagnosed mood or anxiety disorders. Conversely, individuals with stable, well-managed psychiatric diagnoses may have been excluded, potentially limiting generalizability. Effect sizes in both correlation and regression analyses were small, indicating limited explanatory value for most associations. Examination of multiple psychological and sleep variables also raises the risk of Type I error.

Future research using longitudinal designs and neuroimaging, combined with multimodal auditory and psychological assessments, may help clarify how central auditory and limbic networks contribute to tinnitus burden. More granular assessment of emotion regulation—including behavioral tasks or ecological momentary assessment—may also clarify its role. Overall, these results indicate that emotional distress and sleep disturbance are prominent in tinnitus but are not strongly reflected in cochlear electrophysiologic measures.

In conclusion, in this cohort, emotional distress and sleep disturbances were common among tinnitus patients, but cochlear electrophysiologic measures showed only weak and inconsistent associations with psychological or sleep-related outcomes. The results suggest that emotional and sleep-related symptoms in tinnitus are influenced by clinical and psychosocial factors rather than by ECochG or ABR parameters. Further longitudinal and multimodal research is needed to clarify these relationships and guide evidence-based management strategies.

## Data Availability

The raw data supporting the conclusions of this article will be made available by the authors, without undue reservation.
