# Supplementary material for: Associations between cochlear electrophysiology, emotional health, and sleep quality in adults with tinnitus: a comprehensive analysis
Source: Front Psychiatry. 2026 Jan 30;16:1721036. doi: 10.3389/fpsyt.2025.1721036 (PMC12901392; doi:10.3389/fpsyt.2025.1721036)
Supplement: Supplementary file 1 [file Table1.docx]

**Table S1.**  Summary of the cochlear electrophysiologic measures of tinnitus patients

| Variable | Measure | Min | Max |
| --- | --- | --- | --- |
| ECochG readings - no. (%) |  |  |  |
| Normal | 68 (56.67%) | - | - |
| Abnormal | 52 (43.33%) | - | - |
| Otoacoustic emissions (500 Hz) - no. (%) | |  |  |
| No | 68 (56.67%) | - | - |
| Yes | 52 (43.33%) | - | - |
| Auditory Brainstem Response (dBnHL) | |  |  |
| Mean (SD) | 49.49 (12.48) | 20 | 98 |
| SP/AP ratio |  |  |  |
| Mean (SD) | 0.6 (0.20) | 0.1 | 1.1 |
| Wave I latencies (80 dBnHL) |  |  |  |
| Mean (SD) | 2.08 (0.28) | 1.3 | 2.7 |
| Wave III latencies (80 dBnHL) | |  |  |
| Mean (SD) | 4.34 (0.37) | 3.5 | 5.1 |
| Wave V latencies (80 dBnHL) |  |  |  |
| Mean (SD) | 6.00 (0.53) | 4.4 | 7.2 |

no.: frequency; SD: standard deviation; SP/AP: summating potential to action potential ratio; ECochG: electrocochleography;

**Table S2.** A summary of the measures of emotional/psychological characteristics of included patients

| Variable | Measure | Min | Max |
| --- | --- | --- | --- |
| PSS Score |  |  |  |
| Mean (SD) | 31.74 (9.62) | 5 | 40 |
| Observation - no. (%) |  |  |  |
| Stress | 49 (40.83%) | - | - |
| No Stress | 71 (59.17%) | - | - |
| BDI-II Score |  |  |  |
| Mean (SD) | 18.37 (6.02) | 1 | 32 |
| Observation - no. (%) |  |  |  |
| Depressed | 50 (41.67%) | - | - |
| Not Depressed | 70 (58.33%) | - | - |
| GAD-7 Score |  |  |  |
| Mean (SD) | 11.19 (3.98) | 1 | 20 |
| Observation - no. (%) |  |  |  |
| Anxiety | 69 (57.50%) | - | - |
| No Anxiety | 51 (42.50%) | - | - |
| ERQ Score |  |  |  |
| Observation - no. (%) |  |  |  |
| High Cognitive Reappraisal (score 24-42) | 88 (73.33%) | - | - |
| High Expressive Suppression (score 16-28) | 82 (68.33%) | - | - |

PSS: Perceived Stress Scale; BDI-II: Beck Depression Index II; GAD-7: General Anxiety Disorder-7; ERQ: Emotion Regulation Questionnaire.

**Table S3.** A summary of sleep-related parameters and quality of life of tinnitus patients

| Variable | Measure | Min | Max |
| --- | --- | --- | --- |
| PSQI Score |  |  |  |
| Mean (SD) | 13.57 (4.50) | 6 | 20 |
| Observation - no. (%) |  |  |  |
| Poor Sleep Quality | 84 (70%) | - | - |
| Normal Sleep Quality | 36 (30%) | - | - |
| ISI Score |  |  |  |
| Mean (SD) | 18.51 (3.74) | 7 | 24 |
| Observation - no. (%) |  |  |  |
| Insomnia | 49 (40.83%) | - | - |
| No Insomnia | 71 (59.17%) | - | - |
| ESS Score |  |  |  |
| Mean (SD) | 9.04 (1.71) | 5 | 13 |
| Observation - no. (%) |  |  |  |
| Excessive Daytime Sleep | 21 (17.50%) | - | - |
| Normal Daytime Sleep | 99 (82.50%) | - | - |
| Sleep Duration – hour |  |  |  |
| Mean (SD) | 5.03 (1.77) | 1 | 9 |
| Sleep Latency – hour |  |  |  |
| Mean (SD) | 1.45 (0.85) | 0.1 | 3.5 |
| Number of awakenings per night | |  |  |
| Mean (SD) | 1.52 (0.97) | 0 | 3 |
| WHRQoL-BREF |  |  |  |
| Mean (SD) | 67.49 (12.13) | 37 | 100 |

PSQI: Pittsburgh Sleep Quality Index; ISI: Insomnia Severity Scale; ESS: Epworth Sleepiness Sale; WHOQOL-BREF: World Health Organization Quality of Life – BREF; SDSS: Social Functioning Deficit Screening Scale.

**Table S4.** Univariate logistic regression analysis of the predictors of poor sleep quality

| Characteristic | OR | SE | Z | P | 2.5% CI | 97.5% CI |
| --- | --- | --- | --- | --- | --- | --- |
| Age (pr year) | 0.980 | 0.021 | -0.950 | 0.342 | 0.939 | 1.022 |
| Male vs. female | 1.017 | 0.422 | 0.040 | 0.967 | 0.451 | 2.295 |
| Tinnitus duration (per year) | 1.300 | 0.286 | 1.190 | 0.233 | 0.845 | 2.002 |
| Tinnitus severity (THI) - Reference: Slight/no Handicap | | | | | |  |
| Mild | 0.429 | 0.540 | -0.670 | 0.501 | 0.036 | 5.063 |
| Moderate | 0.667 | 0.816 | -0.330 | 0.741 | 0.060 | 7.352 |
| Severe | 1.233 | 1.490 | 0.170 | 0.862 | 0.115 | 13.174 |
| Catastrophic | 0.593 | 0.727 | -0.430 | 0.670 | 0.053 | 6.572 |
| Hearing Threshold (per dB) | 1.024 | 0.018 | 1.340 | 0.180 | 0.989 | 1.059 |
| PTA (dB) | 1.007 | 0.022 | 0.340 | 0.737 | 0.965 | 1.052 |
| ABR (per dBnHL) | 1.005 | 0.016 | 0.320 | 0.752 | 0.974 | 1.037 |
| Otoacoustic Emission - Reference: None | 1.102 | 0.444 | 0.240 | 0.809 | 0.500 | 2.429 |
| SP/AP Ratio (per unit) | 0.227 | 0.230 | -1.460 | 0.143 | 0.031 | 1.655 |
| Wave I Latency (per dBnHL) | 0.545 | 0.391 | -0.850 | 0.397 | 0.134 | 2.220 |
| Wave III Latency (per dBnHL) | 1.173 | 0.621 | 0.300 | 0.763 | 0.416 | 3.313 |
| Wave V Latency (per dBnHL) | 1.214 | 0.461 | 0.510 | 0.609 | 0.577 | 2.554 |
| Frequency (per Hz) | 1.001 | 0.000 | 1.590 | 0.112 | 1.000 | 1.001 |
| Loudness (per dB) | 0.988 | 0.096 | -0.130 | 0.900 | 0.817 | 1.194 |
| Bilateral (vs. unilateral) | 0.813 | 0.367 | -0.460 | 0.646 | 0.335 | 1.970 |
| Hyperacusis | 0.888 | 0.430 | -0.250 | 0.806 | 0.344 | 2.294 |
| Hearing aid | 1.314 | 0.646 | 0.560 | 0.578 | 0.501 | 3.446 |
| Tinnitus masking device | 0.385 | 0.237 | -1.550 | 0.121 | 0.115 | 1.286 |
| Sleep medication | 2.300 | 1.044 | 1.830 | 0.067 | 0.945 | 5.600 |
| Antidepressant | 2.031 | 0.820 | 1.760 | 0.079 | 0.921 | 4.481 |
| anxiolytic | 0.341 | 0.182 | -2.010 | 0.044 | 0.119 | 0.973 |

OR: odds ratio; SE: standard error; CI: confidence interval; ABR: auditory brainstem response; SP/AP: summating potential to action potential; AIC: Akaike’s Information Criterion; BIC: Bayesian Information Criterion.

**Table S5.** Univariate logistic regression analysis of the predictors of insomnia

| Characteristic | OR | SE | Z | P | 2.5% CI | 97.5% CI |
| --- | --- | --- | --- | --- | --- | --- |
| Age (pr year) | 0.994 | 0.020 | -0.320 | 0.751 | 0.956 | 1.033 |
| Male vs. female | 1.476 | 0.581 | 0.990 | 0.323 | 0.682 | 3.193 |
| Tinnitus duration (per year) | 1.174 | 0.235 | 0.800 | 0.424 | 0.793 | 1.739 |
| Tinnitus severity (THI) - Reference: Slight/no Handicap | | | | | |  |
| Mild | 1.800 | 2.277 | 0.460 | 0.642 | 0.151 | 21.477 |
| Moderate | 2.062 | 2.515 | 0.590 | 0.553 | 0.189 | 22.505 |
| Severe | 2.875 | 3.424 | 0.890 | 0.375 | 0.279 | 29.677 |
| Catastrophic | 1.412 | 1.739 | 0.280 | 0.780 | 0.126 | 15.784 |
| Hearing Threshold (per dB) | 1.002 | 0.016 | 0.110 | 0.911 | 0.970 | 1.034 |
| PTA (dB) | 0.956 | 0.021 | -2.070 | 0.039 | 0.916 | 0.998 |
| ABR (per dBnHL) | 1.025 | 0.016 | 1.570 | 0.115 | 0.994 | 1.056 |
| Otoacoustic Emission - Reference: None | 1.474 | 0.553 | 1.040 | 0.301 | 0.707 | 3.075 |
| SP/AP Ratio (per unit) | 0.468 | 0.432 | -0.820 | 0.410 | 0.077 | 2.854 |
| Wave I Latency (per dBnHL) | 0.571 | 0.376 | -0.850 | 0.394 | 0.157 | 2.073 |
| Wave III Latency (per dBnHL) | 0.927 | 0.458 | -0.150 | 0.879 | 0.352 | 2.441 |
| Wave V Latency (per dBnHL) | 0.951 | 0.336 | -0.140 | 0.887 | 0.476 | 1.900 |
| Frequency (per Hz) | 1.000 | 0.000 | 0.760 | 0.445 | 1.000 | 1.001 |
| Loudness (per dB) | 0.969 | 0.087 | -0.350 | 0.724 | 0.812 | 1.156 |
| Bilateral (vs. unilateral) | 0.435 | 0.202 | -1.800 | 0.072 | 0.175 | 1.079 |
| Hyperacusis | 1.447 | 0.654 | 0.820 | 0.414 | 0.596 | 3.511 |
| Hearing aid | 2.989 | 1.629 | 2.010 | 0.045 | 1.027 | 8.699 |
| Tinnitus masking device | 2.200 | 1.359 | 1.280 | 0.202 | 0.655 | 7.386 |
| Sleep medication | 1.005 | 0.447 | 0.010 | 0.991 | 0.420 | 2.403 |
| Antidepressant | 1.830 | 0.700 | 1.580 | 0.114 | 0.864 | 3.874 |
| anxiolytic | 1.209 | 0.513 | 0.450 | 0.654 | 0.526 | 2.777 |

OR: odds ratio; SE: standard error; CI: confidence interval; ABR: auditory brainstem response; SP/AP: summating potential to action potential; AIC: Akaike’s Information Criterion; BIC: Bayesian Information Criterion.

**Table S6.**  Univariate logistic regression analysis of the predictors of stress

| Characteristics | OR | SE | Z | P | 2.5% CI | 97.5% CI |
| --- | --- | --- | --- | --- | --- | --- |
| Age (pr year) | 0.961 | 0.020 | -1.920 | 0.055 | 0.923 | 1.001 |
| Male vs. female | 1.476 | 0.581 | 0.990 | 0.323 | 0.682 | 3.193 |
| Tinnitus duration (per year) | 1.017 | 0.203 | 0.080 | 0.933 | 0.688 | 1.503 |
| Tinnitus severity (THI) - Reference: Slight/no Handicap | | | | | |  |
| Mild | 0.778 | 0.871 | -0.220 | 0.822 | 0.087 | 6.983 |
| Moderate | 0.227 | 0.254 | -1.330 | 0.184 | 0.026 | 2.025 |
| Severe | 0.880 | 0.917 | -0.120 | 0.902 | 0.114 | 6.781 |
| Catastrophic | 1.083 | 1.167 | 0.070 | 0.941 | 0.131 | 8.946 |
| Hearing Threshold (per dB) | 0.997 | 0.016 | -0.180 | 0.857 | 0.966 | 1.029 |
| PTA (dB) | 1.023 | 0.021 | 1.100 | 0.270 | 0.982 | 1.066 |
| ABR (per dBnHL) | 0.997 | 0.015 | -0.180 | 0.857 | 0.968 | 1.027 |
| Otoacoustic Emission - Reference: None | 1.114 | 0.417 | 0.290 | 0.774 | 0.535 | 2.320 |
| SP/AP Ratio (per unit) | 0.919 | 0.843 | -0.090 | 0.927 | 0.152 | 5.548 |
| Wave I Latency (per dBnHL) | 1.751 | 1.162 | 0.840 | 0.399 | 0.477 | 6.430 |
| Wave III Latency (per dBnHL) | 2.511 | 1.282 | 1.800 | 0.071 | 0.923 | 6.829 |
| Wave V Latency (per dBnHL) | 0.905 | 0.320 | -0.280 | 0.776 | 0.453 | 1.808 |
| Frequency (per Hz) | 1.000 | 0.000 | 0.360 | 0.722 | 0.999 | 1.001 |
| Loudness (per dB) | 1.086 | 0.099 | 0.900 | 0.366 | 0.908 | 1.299 |
| Bilateral (vs. unilateral) | 0.792 | 0.345 | -0.540 | 0.592 | 0.338 | 1.858 |
| Hyperacusis | 0.491 | 0.241 | -1.450 | 0.147 | 0.187 | 1.285 |
| Hearing aid | 0.874 | 0.410 | -0.290 | 0.774 | 0.349 | 2.191 |
| Tinnitus masking device | 0.260 | 0.207 | -1.690 | 0.091 | 0.054 | 1.242 |
| Sleep medication | 1.228 | 0.553 | 0.460 | 0.649 | 0.508 | 2.969 |
| Antidepressant | 1.830 | 0.700 | 1.580 | 0.114 | 0.864 | 3.874 |
| anxiolytic | 0.849 | 0.355 | -0.390 | 0.695 | 0.374 | 1.925 |

OR: odds ratio; SE: standard error; CI: confidence interval; ABR: auditory brainstem response; SP/AP: summating potential to action potential; AIC: Akaike’s Information Criterion; BIC: Bayesian Information Criterion.

**Table S7.** Univariate logistic regression analysis of the predictors of anxiety

| Characteristic | OR | SE | Z | P | 2.5% CI | 97.5% CI |
| --- | --- | --- | --- | --- | --- | --- |
| Age (pr year) | 1.030 | 0.021 | 1.450 | 0.146 | 0.990 | 1.071 |
| Male vs. female | 1.290 | 0.496 | 0.660 | 0.507 | 0.608 | 2.739 |
| Tinnitus duration (per year) | 0.979 | 0.194 | -0.100 | 0.917 | 0.664 | 1.445 |
| Tinnitus severity (THI) - Reference: Slight/no Handicap | | | | | |  |
| Mild | 1.800 | 2.277 | 0.460 | 0.642 | 0.151 | 21.477 |
| Moderate | 5.100 | 6.230 | 1.330 | 0.182 | 0.465 | 55.891 |
| Severe | 4.421 | 5.271 | 1.250 | 0.213 | 0.427 | 45.756 |
| Catastrophic | 5.333 | 6.547 | 1.360 | 0.173 | 0.481 | 59.144 |
| Hearing Threshold (per dB) | 0.994 | 0.016 | -0.390 | 0.700 | 0.963 | 1.026 |
| PTA (dB) | 0.982 | 0.020 | -0.900 | 0.371 | 0.943 | 1.022 |
| ABR (per dBnHL) | 0.991 | 0.015 | -0.620 | 0.534 | 0.962 | 1.020 |
| Otoacoustic Emission - Reference: None | 1.341 | 0.503 | 0.780 | 0.434 | 0.643 | 2.795 |
| SP/AP Ratio (per unit) | 0.282 | 0.263 | -1.360 | 0.174 | 0.045 | 1.752 |
| Wave I Latency (per dBnHL) | 0.456 | 0.304 | -1.180 | 0.239 | 0.124 | 1.683 |
| Wave III Latency (per dBnHL) | 2.362 | 1.190 | 1.710 | 0.088 | 0.880 | 6.341 |
| Wave V Latency (per dBnHL) | 0.681 | 0.243 | -1.080 | 0.280 | 0.339 | 1.369 |
| Frequency (per Hz) | 1.000 | 0.000 | 1.300 | 0.193 | 1.000 | 1.001 |
| Loudness (per dB) | 1.037 | 0.093 | 0.410 | 0.682 | 0.870 | 1.237 |
| Bilateral (vs. unilateral) | 1.673 | 0.739 | 1.170 | 0.243 | 0.705 | 3.975 |
| Hyperacusis | 0.615 | 0.278 | -1.070 | 0.283 | 0.254 | 1.492 |
| Hearing aid | 2.018 | 0.947 | 1.500 | 0.134 | 0.805 | 5.063 |
| Tinnitus masking device | 1.541 | 0.990 | 0.670 | 0.501 | 0.438 | 5.428 |
| Sleep medication | 0.911 | 0.405 | -0.210 | 0.834 | 0.381 | 2.175 |
| Antidepressant | 1.133 | 0.422 | 0.340 | 0.737 | 0.546 | 2.351 |
| anxiolytic | 0.754 | 0.319 | -0.670 | 0.505 | 0.329 | 1.729 |

OR: odds ratio; SE: standard error; CI: confidence interval; ABR: auditory brainstem response; SP/AP: summating potential to action potential; AIC: Akaike’s Information Criterion; BIC: Bayesian Information Criterion.

**Table S8.**  Univariate logistic regression analysis of the predictors of depression

| Characteristic | OR | SE | Z | P | 2.5% CI | 97.5% CI |
| --- | --- | --- | --- | --- | --- | --- |
| Age (pr year) | 1.007 | 0.020 | 0.370 | 0.714 | 0.969 | 1.047 |
| Male vs. female | 0.470 | 0.182 | -1.950 | 0.051 | 0.220 | 1.004 |
| Tinnitus duration (per year) | 0.985 | 0.196 | -0.070 | 0.941 | 0.667 | 1.455 |
| Tinnitus severity (THI) - Reference: Slight/no Handicap | | | | | |  |
| Mild | 0.259 | 0.327 | -1.070 | 0.284 | 0.022 | 3.063 |
| Moderate | 0.117 | 0.144 | -1.740 | 0.082 | 0.010 | 1.314 |
| Severe | 0.247 | 0.294 | -1.170 | 0.241 | 0.024 | 2.553 |
| Catastrophic | 0.361 | 0.441 | -0.830 | 0.405 | 0.033 | 3.962 |
| Hearing Threshold (per dB) | 1.007 | 0.016 | 0.410 | 0.681 | 0.975 | 1.039 |
| PTA (dB) | 1.007 | 0.021 | 0.340 | 0.735 | 0.967 | 1.048 |
| ABR (per dBnHL) | 1.038 | 0.017 | 2.300 | 0.021 | 1.006 | 1.072 |
| Otoacoustic Emission - Reference: None | 1.204 | 0.450 | 0.500 | 0.618 | 0.579 | 2.503 |
| SP/AP Ratio (per unit) | 2.322 | 2.145 | 0.910 | 0.362 | 0.380 | 14.201 |
| Wave I Latency (per dBnHL) | 0.962 | 0.627 | -0.060 | 0.952 | 0.268 | 3.454 |
| Wave III Latency (per dBnHL) | 0.636 | 0.315 | -0.910 | 0.362 | 0.241 | 1.681 |
| Wave V Latency (per dBnHL) | 1.011 | 0.356 | 0.030 | 0.974 | 0.507 | 2.017 |
| Frequency (per Hz) | 1.000 | 0.000 | -0.270 | 0.784 | 0.999 | 1.001 |
| Loudness (per dB) | 1.110 | 0.102 | 1.140 | 0.255 | 0.928 | 1.328 |
| Bilateral (vs. unilateral) | 0.757 | 0.329 | -0.640 | 0.522 | 0.323 | 1.774 |
| Hyperacusis | 1.128 | 0.512 | 0.270 | 0.790 | 0.464 | 2.745 |
| Hearing aid | 1.139 | 0.540 | 0.270 | 0.784 | 0.450 | 2.884 |
| Tinnitus masking device | 0.433 | 0.300 | -1.210 | 0.228 | 0.111 | 1.687 |
| Sleep medication | 0.864 | 0.381 | -0.330 | 0.740 | 0.364 | 2.050 |
| Antidepressant | 0.722 | 0.270 | -0.870 | 0.384 | 0.347 | 1.502 |
| anxiolytic | 0.372 | 0.158 | -2.330 | 0.020 | 0.162 | 0.853 |

OR: odds ratio; SE: standard error; CI: confidence interval; ABR: auditory brainstem response; SP/AP: summating potential to action potential; AIC: Akaike’s Information Criterion; BIC: Bayesian Information Criterion.

**Table S9.** Univariate logistic regression analysis of the predictors of high cognitive reappraisal

| Characteristic | OR | SE | Z | P | 2.5% CI | 97.5% CI |
| --- | --- | --- | --- | --- | --- | --- |
| Age (pr year) | 1.014 | 0.022 | 0.660 | 0.512 | 0.972 | 1.059 |
| Male vs. female | 0.758 | 0.334 | -0.630 | 0.528 | 0.320 | 1.796 |
| Tinnitus duration (per year) | 0.779 | 0.173 | -1.120 | 0.263 | 0.504 | 1.206 |
| Tinnitus severity (THI) - Reference: Slight/no Handicap | | | | | |  |
| Mild | 1.238 | 0.843 | 0.310 | 0.755 | 0.325 | 4.706 |
| Moderate | 1.125 | 0.656 | 0.200 | 0.840 | 0.359 | 3.529 |
| Severe | 2.375 | 1.325 | 1.550 | 0.121 | 0.796 | 7.086 |
| Catastrophic | 1.000 | (omitted) | |  |  |  |
| Hearing Threshold (per dB) | 0.999 | 0.018 | -0.060 | 0.953 | 0.964 | 1.035 |
| PTA (dB) | 0.993 | 0.023 | -0.310 | 0.754 | 0.949 | 1.038 |
| ABR (per dBnHL) | 1.009 | 0.017 | 0.540 | 0.587 | 0.976 | 1.043 |
| Otoacoustic Emission - Reference: None | 0.977 | 0.407 | -0.060 | 0.956 | 0.432 | 2.210 |
| SP/AP Ratio (per unit) | 0.901 | 0.919 | -0.100 | 0.919 | 0.122 | 6.655 |
| Wave I Latency (per dBnHL) | 0.303 | 0.233 | -1.550 | 0.121 | 0.067 | 1.370 |
| Wave III Latency (per dBnHL) | 1.022 | 0.561 | 0.040 | 0.968 | 0.349 | 2.997 |
| Wave V Latency (per dBnHL) | 1.228 | 0.483 | 0.520 | 0.601 | 0.568 | 2.655 |
| Frequency (per Hz) | 1.000 | 0.000 | -1.020 | 0.309 | 0.999 | 1.000 |
| Loudness (per dB) | 0.994 | 0.100 | -0.060 | 0.952 | 0.817 | 1.210 |
| Bilateral (vs. unilateral) | 1.625 | 0.832 | 0.950 | 0.343 | 0.595 | 4.435 |
| Hyperacusis | 0.718 | 0.352 | -0.680 | 0.499 | 0.275 | 1.875 |
| Hearing aid | 0.519 | 0.308 | -1.100 | 0.269 | 0.162 | 1.662 |
| Tinnitus masking device | 0.317 | 0.196 | -1.850 | 0.064 | 0.094 | 1.068 |
| Sleep medication | 1.211 | 0.585 | 0.400 | 0.693 | 0.469 | 3.123 |
| Antidepressant | 2.042 | 0.854 | 1.710 | 0.088 | 0.900 | 4.635 |
| anxiolytic | 1.671 | 0.749 | 1.150 | 0.252 | 0.694 | 4.025 |

OR: odds ratio; SE: standard error; CI: confidence interval; ABR: auditory brainstem response; SP/AP: summating potential to action potential; AIC: Akaike’s Information Criterion; BIC: Bayesian Information Criterion.

**Table S10.** Univariate logistic regression analysis of the predictors of high expressive suppression

| Characteristic | OR | SE | Z | P | 2.5% CI | 97.5% CI |
| --- | --- | --- | --- | --- | --- | --- |
| Age (pr year) | 1.023 | 0.021 | 1.070 | 0.282 | 0.982 | 1.066 |
| Male vs. female | 0.637 | 0.269 | -1.070 | 0.286 | 0.278 | 1.459 |
| Tinnitus duration (per year) | 0.883 | 0.186 | -0.590 | 0.556 | 0.585 | 1.335 |
| Tinnitus severity (THI) - Reference: Slight/no Handicap | | | | | |  |
| Mild | 1.729 | 1.164 | 0.810 | 0.416 | 0.462 | 6.468 |
| Moderate | 1.336 | 0.757 | 0.510 | 0.610 | 0.440 | 4.056 |
| Severe | 2.292 | 1.200 | 1.580 | 0.113 | 0.821 | 6.396 |
| Catastrophic | 1.000 | (omitted) | |  |  |  |
| Hearing Threshold (per dB) | 0.989 | 0.017 | -0.620 | 0.534 | 0.956 | 1.024 |
| PTA (dB) | 1.023 | 0.022 | 1.040 | 0.298 | 0.980 | 1.068 |
| ABR (per dBnHL) | 1.008 | 0.016 | 0.480 | 0.629 | 0.977 | 1.040 |
| Otoacoustic Emission - Reference: None | 0.673 | 0.266 | -1.000 | 0.317 | 0.311 | 1.460 |
| SP/AP Ratio (per unit) | 0.829 | 0.804 | -0.190 | 0.846 | 0.124 | 5.548 |
| Wave I Latency (per dBnHL) | 0.548 | 0.386 | -0.850 | 0.393 | 0.138 | 2.181 |
| Wave III Latency (per dBnHL) | 1.167 | 0.609 | 0.300 | 0.767 | 0.420 | 3.246 |
| Wave V Latency (per dBnHL) | 1.278 | 0.479 | 0.650 | 0.512 | 0.613 | 2.663 |
| Frequency (per Hz) | 1.000 | 0.000 | -0.770 | 0.441 | 0.999 | 1.000 |
| Loudness (per dB) | 1.129 | 0.109 | 1.260 | 0.207 | 0.935 | 1.363 |
| Bilateral (vs. unilateral) | 1.726 | 0.838 | 1.130 | 0.261 | 0.667 | 4.469 |
| Hyperacusis | 0.981 | 0.473 | -0.040 | 0.968 | 0.381 | 2.524 |
| Hearing aid | 0.717 | 0.374 | -0.640 | 0.523 | 0.258 | 1.993 |
| Tinnitus masking device | 0.288 | 0.179 | -2.000 | 0.045 | 0.085 | 0.975 |
| Sleep medication | 1.366 | 0.626 | 0.680 | 0.497 | 0.556 | 3.352 |
| Antidepressant | 2.032 | 0.809 | 1.780 | 0.075 | 0.932 | 4.433 |
| anxiolytic | 1.431 | 0.620 | 0.830 | 0.409 | 0.612 | 3.346 |

OR: odds ratio; SE: standard error; CI: confidence interval; ABR: auditory brainstem response; SP/AP: summating potential to action potential; AIC: Akaike’s Information Criterion; BIC: Bayesian Information Criterion.

**Table S11.** Univariate logistic regression analysis of the predictors of excessive daytime sleep

| Characteristic | OR | SE | Z | P | 2.5% CI | 97.5% CI |
| --- | --- | --- | --- | --- | --- | --- |
| Age (pr year) | 0.952 | 0.025 | -1.890 | 0.058 | 0.906 | 1.002 |
| Male vs. female | 0.550 | 0.267 | -1.230 | 0.219 | 0.212 | 1.426 |
| Tinnitus duration (per year) | 0.742 | 0.198 | -1.120 | 0.265 | 0.439 | 1.253 |
| Tinnitus severity (THI) - Reference: Slight/no Handicap | | | | | |  |
| Mild | 0.200 | 0.310 | -1.040 | 0.299 | 0.010 | 4.166 |
| Moderate | 1.500 | 1.837 | 0.330 | 0.741 | 0.136 | 16.542 |
| Severe | 0.615 | 0.750 | -0.400 | 0.690 | 0.057 | 6.700 |
| Catastrophic | 0.261 | 0.357 | -0.980 | 0.327 | 0.018 | 3.824 |
| Hearing Threshold (per dB) | 1.024 | 0.022 | 1.060 | 0.290 | 0.980 | 1.069 |
| PTA (dB) | 1.023 | 0.028 | 0.860 | 0.393 | 0.971 | 1.079 |
| ABR (per dBnHL) | 1.019 | 0.020 | 0.970 | 0.330 | 0.981 | 1.058 |
| Otoacoustic Emission - Reference: None | 1.234 | 0.595 | 0.440 | 0.663 | 0.480 | 3.173 |
| SP/AP Ratio (per unit) | 1.151 | 1.367 | 0.120 | 0.906 | 0.112 | 11.809 |
| Wave I Latency (per dBnHL) | 0.567 | 0.475 | -0.680 | 0.498 | 0.110 | 2.927 |
| Wave III Latency (per dBnHL) | 1.428 | 0.918 | 0.550 | 0.580 | 0.405 | 5.038 |
| Wave V Latency (per dBnHL) | 1.067 | 0.488 | 0.140 | 0.886 | 0.436 | 2.616 |
| Frequency (per Hz) | 0.999 | 0.000 | -1.150 | 0.248 | 0.998 | 1.000 |
| Loudness (per dB) | 0.852 | 0.100 | -1.370 | 0.172 | 0.678 | 1.072 |
| Bilateral (vs. unilateral) | 0.267 | 0.207 | -1.700 | 0.089 | 0.058 | 1.222 |
| Hyperacusis | 1.684 | 0.920 | 0.950 | 0.340 | 0.577 | 4.914 |
| Hearing aid | 1.519 | 1.021 | 0.620 | 0.534 | 0.407 | 5.669 |
| Tinnitus masking device | 2.676 | 1.785 | 1.480 | 0.140 | 0.724 | 9.892 |
| Sleep medication | 0.673 | 0.365 | -0.730 | 0.465 | 0.233 | 1.947 |
| Antidepressant | 0.811 | 0.391 | -0.440 | 0.663 | 0.315 | 2.084 |
| anxiolytic | 0.890 | 0.476 | -0.220 | 0.828 | 0.312 | 2.537 |

OR: odds ratio; SE: standard error; CI: confidence interval; ABR: auditory brainstem response; SP/AP: summating potential to action potential; AIC: Akaike’s Information Criterion; BIC: Bayesian Information Criterion.

**Table S12.**  Univariate linear regression analysis of the predictors of quality-of-life score (WHOQoL-BREF)

| Characteristic | Coefficient | SE | Z | P | 2.5% CI | 97.5% CI |
| --- | --- | --- | --- | --- | --- | --- |
| Age (pr year) | -0.208 | 0.117 | -1.780 | 0.077 | -0.439 | 0.023 |
| Male vs. female | 0.875 | 2.320 | 0.380 | 0.707 | -3.718 | 5.468 |
| Tinnitus duration (per year) | -2.624 | 1.170 | -2.240 | 0.027 | -4.942 | -0.307 |
| Tinnitus severity (THI) - Reference: Slight/no Handicap | | | | | |  |
| Mild | -2.000 | 6.847 | -0.290 | 0.771 | -15.563 | 11.563 |
| Moderate | -3.463 | 6.562 | -0.530 | 0.599 | -16.462 | 9.536 |
| Severe | -1.606 | 6.379 | -0.250 | 0.802 | -14.243 | 11.031 |
| Catastrophic | -5.940 | 6.596 | -0.900 | 0.370 | -19.006 | 7.126 |
| Hearing Threshold (per dB) | -0.028 | 0.097 | -0.290 | 0.772 | -0.221 | 0.164 |
| PTA (dB) | -0.118 | 0.122 | -0.960 | 0.339 | -0.360 | 0.125 |
| ABR (per dBnHL) | 0.000 | 0.089 | 0.000 | 1.000 | -0.177 | 0.177 |
| Otoacoustic Emission - Reference: None | 0.897 | 2.244 | 0.400 | 0.690 | -3.547 | 5.341 |
| SP/AP Ratio (per unit) | 1.992 | 5.493 | 0.360 | 0.718 | -8.886 | 12.870 |
| Wave I Latency (per dBnHL) | 0.045 | 3.921 | 0.010 | 0.991 | -7.720 | 7.810 |
| Wave III Latency (per dBnHL) | 2.179 | 2.952 | 0.740 | 0.462 | -3.666 | 8.024 |
| Wave V Latency (per dBnHL) | 1.590 | 2.111 | 0.750 | 0.453 | -2.591 | 5.770 |
| Frequency (per Hz) | 0.000 | 0.002 | 0.170 | 0.866 | -0.004 | 0.005 |
| Loudness (per dB) | 0.701 | 0.536 | 1.310 | 0.193 | -0.360 | 1.762 |
| Bilateral (vs. unilateral) | -0.967 | 2.569 | -0.380 | 0.707 | -6.053 | 4.120 |
| Hyperacusis | -1.076 | 2.739 | -0.390 | 0.695 | -6.499 | 4.347 |
| Hearing aid | 0.017 | 2.827 | 0.010 | 0.995 | -5.582 | 5.616 |
| Tinnitus masking device | -1.194 | 3.708 | -0.320 | 0.748 | -8.537 | 6.149 |
| Sleep medication | 0.395 | 2.665 | 0.150 | 0.882 | -4.882 | 5.673 |
| Antidepressant | -0.626 | 2.245 | -0.280 | 0.781 | -5.072 | 3.820 |
| anxiolytic | 2.247 | 2.508 | 0.900 | 0.372 | -2.720 | 7.214 |

SE: standard error; CI: confidence interval; ABR: auditory brainstem response; SP/AP: summating potential to action potential; AIC: Akaike’s Information Criterion; BIC: Bayesian Information Criterion.
